# Structure of a diorganotelluroxonium(IV) cation, {[2,6-(CH_2_NMe_2_)_2_C_6_H_3_Te(μ-O)]_2_}^2+^, with the tri­chlorido­(dimethyl sulfoxide)­platinum(II) anion

**DOI:** 10.1107/S2056989020011482

**Published:** 2020-08-28

**Authors:** Anand Gupta, Rajesh Deka, Ray J. Butcher, Harkesh B. Singh

**Affiliations:** a Department of Chemistry, Indian Institute of Technology Bombay, Powai, Mumbai 400 076, India; b Department of Chemistry, Howard University, 525 College Street NW, Washington DC 20059, USA

**Keywords:** crystal structure, heteroleptic diorganotelluride, diorganotelluroxonium(IV) cation

## Abstract

In the title salt, di-μ-oxido-bis­{2,6-bis­[(di­methyl­amino)­meth­yl]phenyl-κ*C* ^1^}tellurium(­IV) bis[tri­chlorido­(dimethyl sulfoxide-κ*S*)platinate(II)], (C_24_H_38_N_4_O_2_Te_2_)[PdCl_3_(C_2_H_6_OS)]_2_, which crystallizes in the triclinic space group *P* , each Te atom is in a distorted five-coordinated TeO_2_N_2_C square-pyramidal geometry (τ values of 0.026 and 0.001) with the C atoms of the phenyl rings occupying the apical positions. The phenyl rings in the [C_24_H_38_N_4_O_2_Te_2_]^2+^ cation are in a *cis* arrangement to enable this species to participate in Te⋯Cl cation–anion inter­actions. There are also C—H⋯O inter­actions involving the dimethyl sulfoxide ligands and numerous cation–anion and anion–anion C—H⋯Cl inter­actions, which link the ions into a complex three-dimensional array.

## Abstract 2

In the structure of the salt [C_24_H_38_N_4_O_2_Te_2_]^2+^ [PdCl_3_(DMSO)]^−^_2_, the phenyl rings in the [C_24_H_38_N_4_O_2_Te_2_]^2+^ cation are in a *cis* arrangement to enable this species to participate in Te⋯Cl cation–anion inter­actions.

## Chemical context   

After the initial discovery (Moulton & Shaw, 1976 ▸) and seminal contributions from various research groups, the coordination chemistry of pincer ligands has become an important field in coordination chemistry (Peris & Crabtree, 2018 ▸). One pincer ligand scaffold that has recently attracted considerable attention with respect to its inter­esting structural features and reactivity, is the NCN pincer ligand, [2,6-(Me_2_NCH_2_)_2_C_6_H_3_] (**HL**).
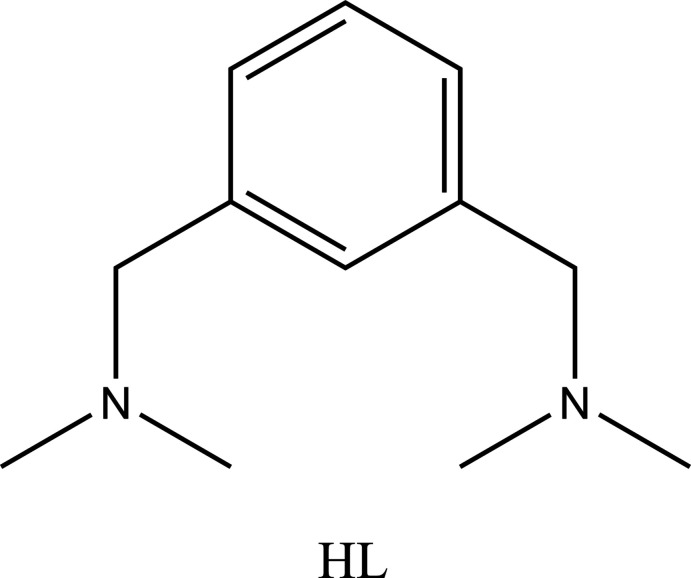


Of particular inter­est are the group 16 derivatives of these ligands where, due to the presence of intra­molecular N→*M* inter­actions from the two coordinating auxiliary arms, their compounds show inter­esting reactivity and have been used in the formation of selenium cations (Fujihara *et al.*, 1995 ▸; Poleschner & Seppelt, 2004 ▸, 2013 ▸; Gupta *et al.*, 2017 ▸; Pop *et al.*, 2014 ▸; Varga *et al.*, 2010 ▸; Rani *et al.*, 2018 ▸). It is worth noting that, compared to the selenenium cation of ligand **L**, studies on their higher congener *i.e*., tellurenium cations, are relatively scarce in the literature and this was the initial impetus for this work. Furukawa and co-workers reported the synthesis of a tellurenium cation by the reaction of heteroleptic diorganotelluride **L**Te*R* (where *R* = *n*-but­yl) with Br_2_/K[PF6] (Fujihara *et al.*, 1995 ▸). However, the structural elucidation of the tellurenium cation of the ligand **L** remained elusive until Silvestru and co-workers reported the first structural characterization of a tellurenium cation (Beleaga *et al.*, 2011 ▸).

It is inter­esting to note that the related tin(II) cations of ligand **L**, containing one lone pair of electrons, have been used as ligands to isolate heterobimetallic complexes **4a**,**b** (Martincová *et al.*, 2011 ▸, 2012 ▸). However, no such coordination chemistry has been explored for the selenenium(II) and tellurenium(II) cations of ligand **L**, which have two such pairs of electrons. A notable work is that by Lin & Gabbaï (2013 ▸) where they used Te^IV^ cations having one lone pair of electrons as ligands for isolating complex **5** where the Te^IV^ center acted as a σ-acceptor (*Z*-type) ligand.
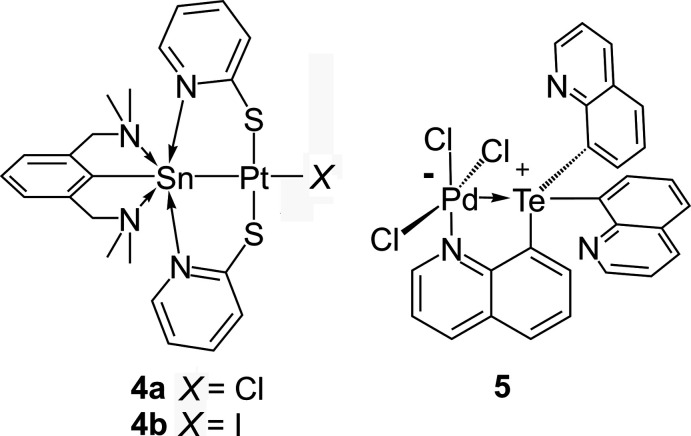


Recently, we investigated the reactivity of the homoleptic diorganotelluride [2,6 (Me_2_NCH_2_)_2_C_6_H_3_]_2_Te, **1** with SO_2_Cl_2_:K_2_PdCl_4_ (Gupta *et al.*, 2019 ▸). We observed that the diorganotelluride underwent intra­molecular chalcogen bonding (IChB) mediated Te-de­aryl­ation to afford the first example of a Pd^II^ complex [2,6(Me_2_NCH_2_)_2_C_6_H_3_]TePdCl_3_, with any organotellurenium(II) cation as a ligand. This might be due to the formation of the highly stable tellurenium(II) cation where the Te is T-shaped and involved in a three-centered, four-electron bond. While checking the reproduc­ibility of the reaction, in one instance, because of the adventitious uptake of oxygen, the reaction unexpectedly resulted in the isolation of the title compound, which contains the ditelluroxonium(IV) cation **2**, [2,6-(CH_2_NMe_2_)_2_C_6_H_3_Te(μ-O)]_2_ with the PdCl_3_(DMSO) anion. It is worth noting that Furukawa and coworkers have reported a similar diorganotelluroxonium(IV) cation namely, [2,6-(CH_2_NMe_2_)_2_C_6_H_3_Te(μ-O)]_2_·PF_6_, by the reaction of the diorganotelluride [2,6 (Me_2_NCH_2_)_2_C_6_H_3_]_2_Te with the oxidizing agent NOPF_6_ (Kobayashi *et al.*, 2000 ▸).
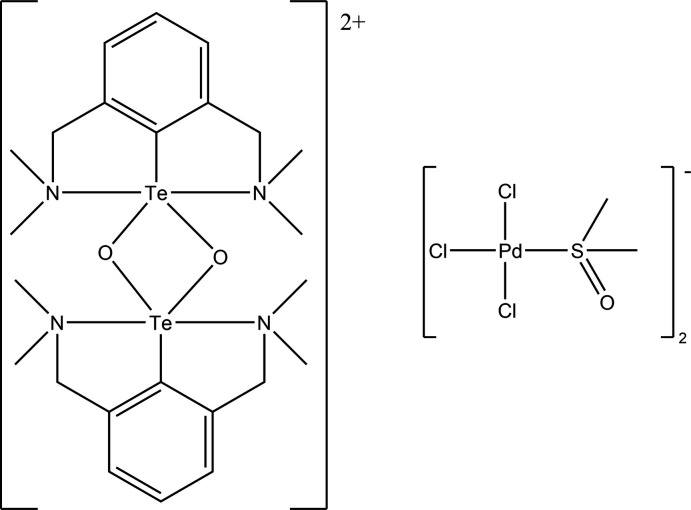


## Structural commentary   

**Figure 1:**
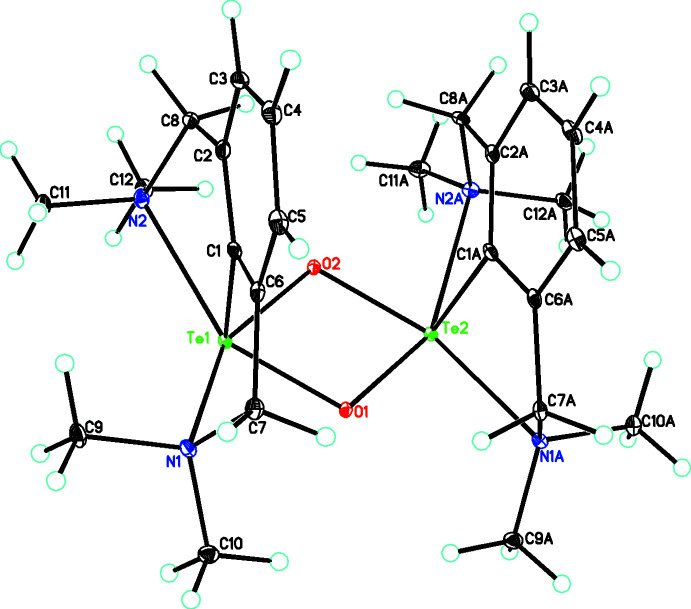
The mol­ecular structure of the [C_24_H_38_N_4_O_2_Te_2_]^2+^ cation, showing the *cis* arrangement of the phenyl rings with respect to the Te_2_O_2_ core. Atomic displacement parameters are drawn at the 30% probability level.

**Figure 2:**
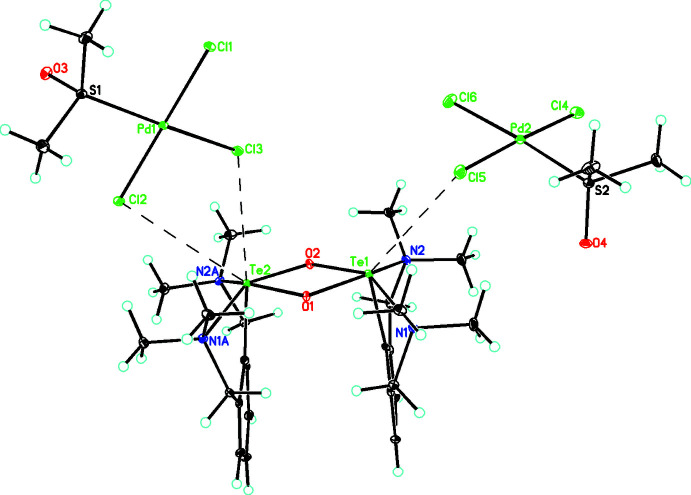
Diagram of the [C_24_H_38_N_4_O_2_Te_2_]^2+^ anion and the [PdCl_3_(DMSO)]^−^ anions linked by Te⋯Cl inter­actions (shown as dashed lines).

The title structure represents a rare example of a structurally characterized diorganotelluroxonium(IV) cation and key geometrical data are listed in Table 1 ▸. The salt [C_24_H_38_N_4_O_2_Te_2_]^2+^ ·[PdCl_3_(DMSO)]^−^_2_, **3**, crystallizes in the triclinic space group *P* 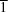. In the structure of the cation **2** there is a *cis* arrangement of the aryl rings of the attached 2,6-[(di­methyl­amino)­meth­yl)]phenyl substituents (Fig. 1 ▸). This is in contrast to that observed in the structure of [2,6-(CH_2_NMe_2_)_2_C_6_H_3_Te(μ-O)]_2_^+^·PF_6_^−^, wherein the cation lies on a center of inversion and thus the aryl groups are in a *trans* configuration (Kobayashi *et al.*, 2000 ▸). Each Te atom is in a five-coordinate geometry with the phenyl rings occupying the apical position. An analysis of this geometry using the continuous shape measurement (CSM) method (Cirera *et al.*, 2005 ▸; Llunell *et al.*, 2013 ▸) and using the four appropriate reference shapes [vacant octa­hedron, *C_4v_*; trigonal bipyramid, *D_3h_*; square pyramid, *C_4v_*; and Johnson trigonal bipyramid, *D_3h_*] showed that the closest fit was the vacant octa­hedron. The Te—N bond distances, lying in the range from 2.450 (2)–2.495 (2) Å for **2**, are in good agreement with the values observed in [2,6-(CH_2_NMe_2_)_2_C_6_H_3_Te(μ-O)]_2_^+^·PF_6_^−^ [2.475 (5)–2.486 (5) Å] (Kobayashi *et al.*, 2000 ▸). In **2**, the dihedral angle between the two aryl groups is 6.2 (2)° and those between the Te_2_O_2_ plane and the aryl rings are 88.77 (8) and 85.00 (8)°, indicating that the two aryl groups are not coplanar, and are too far apart to form π–π stacking inter­actions (the closest contact is between C1 and C1*A* at 3.672 Å). Thus, the driving force for the adoption of this sterically unfavorable *cis* conformation appears to be the formation of Te⋯Cl cation–anion inter­actions, which would not be possible if the *trans* conformation were adopted. In this case, there is a short Te2⋯Cl3 contact of 3.386 (1) Å and longer contacts of 3.833 (1) Å (Te2⋯Cl2) and 3.991 (1) Å (Te1⋯Cl5) (see Fig. 2 ▸). In contrast, in the case of [2,6-(CH_2_NMe_2_)_2_C_6_H_3_Te(μ-O)]_2_^+^·PF_6_^−^, no such cation–anion inter­actions are present and hence the more sterically favorable *trans* conformation is adopted. In the other two related structures containing the Te_2_O_2_^2+^ core dication, the same *cis* configuration is adopted to allow the formation of inter­ionic Te⋯O inter­actions (Hupf *et al.*, 2017 ▸; Deka *et al.*, 2020 ▸).

## Supra­molecular features   

**Figure 3:**
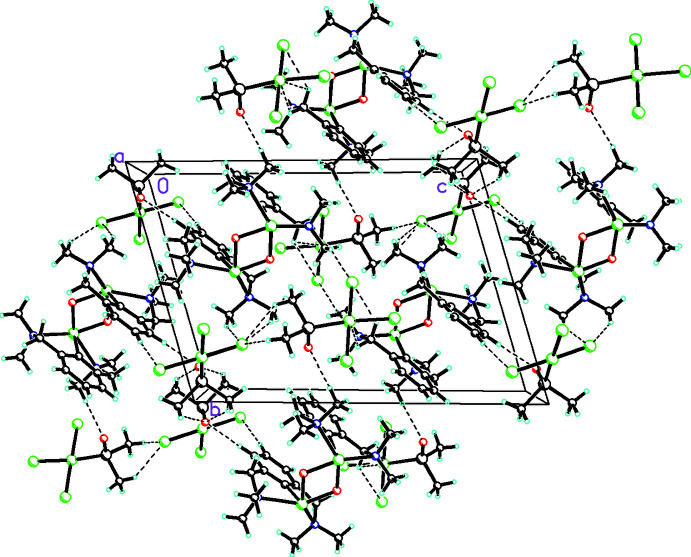
Packing diagram showing how the Te⋯Cl cation–anion inter­actions, C—H⋯O inter­actions involving the DMSO ligands, and numerous cation–anion and anion–anion C—H⋯Cl inter­actions linking these moieties into a complex three-dimensional array (all inter­actions shown as dashed lines).

In addition to the Te⋯Cl cation–anion inter­actions mentioned above, there are also C—H⋯O inter­actions involving the DMSO ligands and numerous cation–anion and anion–anion C—H⋯Cl inter­actions (Table 2 ▸), which link the ions into a complex three-dimensional array, as seen in Fig. 3 ▸.

## Database survey   

There are only three reports available containing a cation with the Te_2_O_2_^2+^ core. The first report on the mol­ecular structure of a diorganotelluroxonium(IV) cation was made by Furukawa and co-workers (Kobayashi *et al.*, 2000 ▸; Cambridge Structural Database refcode XAGGER), which contains a cation [2,6-(CH_2_NMe_2_)_2_C_6_H_3_Te(μ-O)]_2_^+^ charge-balanced as the PF_6_^−^ salt. Beckmann and coworkers reported the mol­ecular structure of [(6-Ph_2_P(O)-Ace-5-) Te(μ-O)]_2_·2OTf [Ace = acenaphthyl; Hupf *et al.*, 2017 ▸; refcode CAZCEO). Recently, we have reported the third example of a structurally characterized ditelluroxonium cation, namely [ppyTe(μ-O)]_2_·2ClO_4_ (ppy = phenyl­pyridine), stabilized by extensive IChB inter­actions (Deka *et al.*, 2020 ▸; refcode PUBWAN).

## Synthesis and crystallization   

To a solution of **1** (0.10 g, 0.20 mmol) in CCl_4_ (3 ml), a solution of SO_2_Cl_2_ (0.03 g, 17.76 µL, 0.22 mmol) in CCl_4_ (2 ml) was added dropwise at 273 K under an N_2_ atmosphere. After stirring the reaction mixture for 1 h, hexane (10 ml) was added, resulting in the formation of a white precipitate. The precipitate was washed with hexane (2 × 5 ml) and dissolved in THF (20 ml). To it, K_2_PdCl_4_ (0.06 g, 0.20 mmol) and KOH (0.01 g, 0.17 mmol) were added at ambient temperature. After stirring for 12 h, the solvent was removed under vacuum, resulting in the precipitation of a dark-purple solid. The solid was washed with CH_2_Cl_2_ (3 × 5 ml) and Et_2_O (2 × 10 ml), and dried under vacuum to afford the analytically pure solid of **2**. Dark-purple prisms of **2** suitable for single-crystal diffraction analysis were acquired by slow diffusion of Et_2_O into a DMSO solution at room temperature.
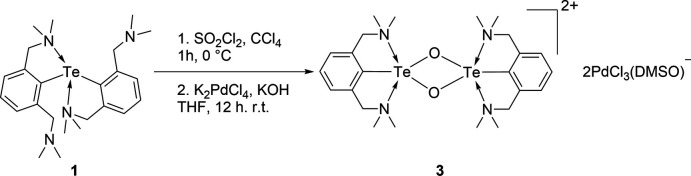


Yield: 59%; m.p. 444–446 K; ^1^H NMR: δ (ppm) 7.35–7.31 (*m*, 1H, Ar-H), 7.24–7.20 (*m*, 2H, Ar-H), 4.25 (*d*, 2H, ArCH_2_), 3.55 (*d*, 2H, ArCH_2_), 2.52 (*s*, 6H, NMe_2_), 2.41 (*s*, 6H, NMe_2_); ^13^C NMR: δ (ppm) 130.55, 125.22, 122.89, 120.44, 67.14, 45.70; ^125^Te NMR: δ (ppm) 1500; ESI–MS (positive mode): *m*/*z* calculated for [C_12_H_19_N_2_OTe]^+^: 336.0545, found: 336.0541.

## Refinement   

Crystal data, data collection and structure refinement details are summarized in Table 3 ▸. A riding model was used for the H atoms with atomic displacement parameters = 1.2*U*_eq_(C) [1.5*U*_eq_(CH_3_)] and C—H distances ranging from 0.95 to 0.99 Å.

## Supplementary Material

Crystal structure: contains datablock(s) I. DOI: 10.1107/S2056989020011482/hb7941sup1.cif

Structure factors: contains datablock(s) I. DOI: 10.1107/S2056989020011482/hb7941Isup2.hkl

CCDC reference: 1563166

Additional supporting information:  crystallographic information; 3D view; checkCIF report

## Figures and Tables

**Table 1:** Selected geometric parameters (Å, °)

Te1—O1	1.9836 (19)	Te2—O1	2.0071 (19)
Te1—O2	1.9871 (19)	Te2—O2	1.9844 (18)
Te1—C1	2.089 (3)	Te2—N1*A*	2.477 (2)
Te1—N2	2.450 (2)	Te2—N2*A*	2.459 (2)
Te1—N1	2.495 (2)	Te2—C1*A*	2.107 (3)
			
O1—Te1—O2	76.92 (8)	O2—Te2—O1	76.44 (8)
O1—Te1—C1	96.99 (10)	O2—Te2—C1*A*	95.66 (10)
O2—Te1—C1	93.43 (10)	O1—Te2—C1*A*	94.79 (10)
O1—Te1—N2	150.20 (8)	O2—Te2—N2*A*	76.19 (8)
O2—Te1—N2	75.53 (8)	O1—Te2—N2*A*	148.65 (8)
C1—Te1—N2	73.66 (10)	C1*A*—Te2—N2*A*	73.06 (10)
O1—Te1—N1	77.12 (8)	O2—Te2—N1*A*	148.72 (7)
O2—Te1—N1	148.63 (8)	O1—Te2—N1*A*	75.46 (8)
C1—Te1—N1	72.64 (10)	C1*A*—Te2—N1*A*	73.37 (10)
N2—Te1—N1	124.18 (8)	N2*A*—Te2—N1*A*	125.35 (9)

**Table 2:** Hydrogen-bond geometry (Å, °)

*D*—H⋯*A*	*D*—H	H⋯*A*	*D*⋯*A*	*D*—H⋯*A*
C7—H7*A*⋯Cl1	0.99	2.99	3.768 (3)	136
C7*A*—H7*AB*⋯Cl1	0.99	2.90	3.663 (3)	135
C9*A*—H9*AB*⋯Cl1^i^	0.98	2.85	3.697 (4)	145
C9*A*—H9*AC*⋯O1	0.98	2.49	3.000 (3)	112
C9—H9*B*⋯O4^ii^	0.98	2.64	3.355 (4)	130
C9—H9*B*⋯Cl5^ii^	0.98	2.87	3.741 (4)	149
C10*A*—H10*C*⋯Cl2^iii^	0.98	2.80	3.706 (3)	154
C10—H10*E*⋯O1	0.98	2.57	3.078 (4)	112
C10—H10*F*⋯Cl5^ii^	0.98	2.82	3.711 (4)	151
C11*A*—H11*E*⋯O2	0.98	2.56	3.048 (4)	110
C11*A*—H11*F*⋯Cl3^iii^	0.98	2.91	3.639 (4)	132
C12*A*—H12*A*⋯O3^iv^	0.98	2.42	3.316 (4)	151
C12*A*—H12*A*⋯Cl2^iv^	0.98	2.97	3.595 (3)	123
C12*A*—H12*B*⋯Cl2^iii^	0.98	2.80	3.678 (4)	150
C12—H12*D*⋯Cl6^v^	0.98	2.92	3.825 (3)	155
C12—H12*F*⋯O2	0.98	2.43	2.980 (4)	115
C21—H21*A*⋯Cl5^iv^	0.98	2.77	3.628 (3)	147
C21—H21*C*⋯Cl3^vi^	0.98	2.59	3.506 (3)	157
C22—H22*B*⋯Cl5^iv^	0.98	2.84	3.681 (4)	144
C22—H22*C*⋯Cl1^vi^	0.98	2.86	3.670 (4)	141
C22—H22*D*⋯Cl2	0.98	2.77	3.308 (4)	115
C31—H31*B*⋯O4^vii^	0.98	2.53	3.389 (4)	147
C31—H31*C*⋯Cl6^v^	0.98	2.94	3.796 (4)	146
C32—H32*B*⋯O4^vii^	0.98	2.46	3.336 (4)	149
C32—H32*C*⋯Cl5	0.98	2.73	3.275 (4)	115
C32—H32*D*⋯Cl4^v^	0.98	2.58	3.541 (4)	167

Symmetry codes: (i) 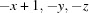; (ii) 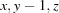; (iii) 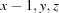; (iv) 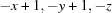; (v) 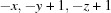; (vi) 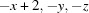; (vii) 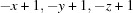.

**Table 3:** Experimental details

Crystal data	
Chemical formula	(C_24_H_38_N_4_O_2_Te_2_)[PdCl_3_(C_2_H_6_OS)]_2_
*M*_r_	1251.54
Crystal system, space group	Triclinic, *P* 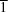
Temperature (K)	100
*a*, *b*, *c* (Å)	9.6333 (19), 12.770 (3), 17.956 (4)
α, β, γ (°)	73.82 (3), 83.55 (3), 88.86 (3)
*V* (Å^3^)	2107.8 (8)
*Z*	2
Radiation type	Mo *K*α
μ (mm^−1^)	2.73
Crystal size (mm)	0.39 × 0.20 × 0.14

Data collection	
Diffractometer	Rigaku Saturn 724 Dual Source CCD
Absorption correction	Numerical (*NUMABS*; Rigaku, 1999 ▸)
*T*_min_, *T*_max_	0.417, 0.703
No. of measured, independent and observed [*I* > 2σ(*I*)] reflections	16345, 7532, 6351
*R*_int_	0.028
(sin θ/λ)_max_ (Å^−1^)	0.602

Refinement	
*R*[*F* ^2^ > 2σ(*F* ^2^)], *wR*(*F* ^2^), *S*	0.022, 0.052, 0.99
No. of reflections	7532
No. of parameters	445
H-atom treatment	H-atom parameters constrained
Δρ_max_, Δρ_min_ (e Å^−3^)	0.72, −0.95

Computer programs: *CrystalClear-SM Expert* (Rigaku, 2012 ▸), *SHELXT* (Sheldrick, 2015*a* ▸), *SHELXL2018/3* (Sheldrick, 2015*b* ▸) and *SHELXTL* (Sheldrick, 2008 ▸).

## supplementary crystallographic information

### Crystal data

**Table:**

(C_24_H_38_N_4_O_2_Te_2_)[PdCl_3_(C_2_H_6_OS)]_2_	*Z* = 2
*M**_r_* = 1251.54	*F*(000) = 1216
Triclinic, *P*1	*D*_x_ = 1.972 Mg m^−^^3^
*a* = 9.6333 (19) Å	Mo *K*α radiation, λ = 0.71073 Å
*b* = 12.770 (3) Å	Cell parameters from 4389 reflections
*c* = 17.956 (4) Å	θ = 3.0–25.3°
α = 73.82 (3)°	µ = 2.73 mm^−^^1^
β = 83.55 (3)°	*T* = 100 K
γ = 88.86 (3)°	Prism, dark purple
*V* = 2107.8 (8) Å^3^	0.39 × 0.20 × 0.14 mm

### Data collection

**Table:**

Rigaku Saturn 724 Dual Source CCD diffractometer	6351 reflections with *I* > 2σ(*I*)
Detector resolution: 28.5714 pixels mm^-1^	*R*_int_ = 0.028
ω scans	θ_max_ = 25.4°, θ_min_ = 3.0°
Absorption correction: numerical (NUMABS; Rigaku, 1999)	*h* = −10→11
*T*_min_ = 0.417, *T*_max_ = 0.703	*k* = −15→15
16345 measured reflections	*l* = −18→21
7532 independent reflections	

### Refinement

**Table:**

Refinement on *F*^2^	Primary atom site location: structure-invariant direct methods
Least-squares matrix: full	Secondary atom site location: difference Fourier map
*R*[*F*^2^ > 2σ(*F*^2^)] = 0.022	Hydrogen site location: inferred from neighbouring sites
*wR*(*F*^2^) = 0.052	H-atom parameters constrained
*S* = 0.99	*w* = 1/[σ^2^(*F*_o_^2^) + (0.0225*P*)^2^] where *P* = (*F*_o_^2^ + 2*F*_c_^2^)/3
7532 reflections	(Δ/σ)_max_ = 0.001
445 parameters	Δρ_max_ = 0.72 e Å^−^^3^
0 restraints	Δρ_min_ = −0.95 e Å^−^^3^

### Special details

**Table:**

Geometry. All esds (except the esd in the dihedral angle between two l.s. planes) are estimated using the full covariance matrix. The cell esds are taken into account individually in the estimation of esds in distances, angles and torsion angles; correlations between esds in cell parameters are only used when they are defined by crystal symmetry. An approximate (isotropic) treatment of cell esds is used for estimating esds involving l.s. planes.

### Fractional atomic coordinates and isotropic or equivalent isotropic displacement parameters (Å^2^)

**Table:**

	*x*	*y*	*z*	*U*_iso_*/*U*_eq_	
Te1	0.33401 (2)	0.04241 (2)	0.28514 (2)	0.01035 (6)	
Pd1	0.94231 (2)	0.16499 (2)	0.01042 (2)	0.01127 (6)	
Cl1	0.81302 (7)	0.00483 (6)	0.05078 (4)	0.01552 (17)	
S1	0.89105 (8)	0.19555 (6)	−0.11264 (4)	0.01283 (17)	
N1A	0.4790 (2)	0.2407 (2)	0.02780 (13)	0.0115 (6)	
C1A	0.4649 (3)	0.3347 (2)	0.14904 (16)	0.0127 (7)	
O1	0.40044 (19)	0.09584 (16)	0.17231 (10)	0.0107 (5)	
N1	0.5460 (2)	−0.0692 (2)	0.27189 (14)	0.0133 (6)	
C1	0.4960 (3)	0.1100 (2)	0.32559 (16)	0.0112 (6)	
Te2	0.29877 (2)	0.23691 (2)	0.13837 (2)	0.00938 (5)	
Pd2	0.10998 (2)	0.68021 (2)	0.44760 (2)	0.01459 (6)	
S2	0.29029 (8)	0.56851 (7)	0.47852 (4)	0.01472 (17)	
N2	0.2358 (2)	0.0782 (2)	0.40747 (13)	0.0144 (6)	
O2	0.23723 (19)	0.18393 (17)	0.25186 (10)	0.0114 (5)	
N2A	0.1943 (2)	0.3983 (2)	0.17064 (14)	0.0129 (6)	
C2A	0.4399 (3)	0.4105 (2)	0.19095 (16)	0.0115 (7)	
C2	0.4621 (3)	0.1733 (3)	0.37634 (16)	0.0147 (7)	
Cl3	0.96704 (8)	0.15286 (7)	0.14002 (4)	0.01622 (17)	
O3	0.7830 (2)	0.27971 (18)	−0.13078 (11)	0.0175 (5)	
C3A	0.5517 (3)	0.4697 (3)	0.20169 (17)	0.0158 (7)	
H3AA	0.537194	0.519754	0.232222	0.019*	
C3	0.5686 (3)	0.2270 (3)	0.39672 (17)	0.0176 (7)	
H3A	0.548200	0.269940	0.432012	0.021*	
Cl2	1.07137 (8)	0.32486 (6)	−0.02994 (4)	0.01833 (17)	
Cl4	0.00771 (8)	0.63088 (7)	0.57463 (4)	0.02292 (19)	
O4	0.4201 (2)	0.62606 (18)	0.48098 (12)	0.0172 (5)	
C4A	0.6855 (3)	0.4554 (3)	0.16736 (17)	0.0173 (7)	
H4AA	0.762485	0.494414	0.175776	0.021*	
C4	0.7059 (3)	0.2181 (3)	0.36546 (17)	0.0187 (7)	
H4A	0.777779	0.259323	0.376730	0.022*	
Cl5	0.21996 (8)	0.73154 (8)	0.32293 (4)	0.0282 (2)	
C5	0.7392 (3)	0.1501 (3)	0.31822 (17)	0.0160 (7)	
H5A	0.833870	0.142102	0.299431	0.019*	
C5A	0.7069 (3)	0.3844 (3)	0.12092 (17)	0.0149 (7)	
H5AA	0.797667	0.377838	0.095907	0.018*	
Cl6	−0.06703 (9)	0.80252 (8)	0.40930 (5)	0.0279 (2)	
C6A	0.5978 (3)	0.3236 (3)	0.11093 (16)	0.0125 (7)	
C6	0.6342 (3)	0.0934 (3)	0.29826 (16)	0.0132 (7)	
C7	0.6628 (3)	0.0113 (3)	0.25245 (17)	0.0147 (7)	
H7A	0.672555	0.048960	0.195871	0.018*	
H7B	0.751358	−0.026580	0.265278	0.018*	
C7A	0.6129 (3)	0.2492 (3)	0.05866 (16)	0.0125 (7)	
H7AA	0.686396	0.278106	0.014850	0.015*	
H7AB	0.641317	0.175920	0.088484	0.015*	
C8A	0.2900 (3)	0.4297 (3)	0.21981 (16)	0.0132 (7)	
H8AA	0.268099	0.386007	0.274743	0.016*	
H8AB	0.277568	0.507681	0.217519	0.016*	
C8	0.3110 (3)	0.1770 (3)	0.40978 (17)	0.0156 (7)	
H8A	0.306764	0.180806	0.464321	0.019*	
H8B	0.266195	0.242929	0.379013	0.019*	
C9A	0.4723 (3)	0.1439 (3)	−0.00092 (17)	0.0166 (7)	
H9AA	0.542769	0.150699	−0.045863	0.025*	
H9AB	0.379207	0.138039	−0.016649	0.025*	
H9AC	0.490338	0.078522	0.040633	0.025*	
C9	0.5562 (3)	−0.1486 (3)	0.34914 (17)	0.0191 (8)	
H9A	0.639146	−0.193593	0.346083	0.029*	
H9B	0.472556	−0.195482	0.364070	0.029*	
H9C	0.563665	−0.109263	0.388236	0.029*	
C10A	0.4566 (3)	0.3395 (3)	−0.03633 (16)	0.0160 (7)	
H10A	0.527925	0.343701	−0.080528	0.024*	
H10B	0.463240	0.404439	−0.017966	0.024*	
H10C	0.363715	0.335566	−0.052866	0.024*	
C10	0.5437 (3)	−0.1274 (3)	0.21195 (17)	0.0168 (7)	
H10D	0.629019	−0.170147	0.210198	0.025*	
H10E	0.538531	−0.074539	0.160977	0.025*	
H10F	0.462038	−0.176280	0.224740	0.025*	
C11	0.2664 (3)	−0.0165 (3)	0.47220 (17)	0.0203 (8)	
H11A	0.229057	−0.003978	0.522014	0.030*	
H11B	0.367714	−0.026302	0.470967	0.030*	
H11C	0.222739	−0.082175	0.466593	0.030*	
C11A	0.0504 (3)	0.3825 (3)	0.21053 (18)	0.0187 (8)	
H11D	0.016516	0.451347	0.219420	0.028*	
H11E	0.049883	0.326727	0.260701	0.028*	
H11F	−0.010523	0.358949	0.177925	0.028*	
C12A	0.1962 (3)	0.4811 (3)	0.09374 (17)	0.0172 (7)	
H12A	0.167029	0.551598	0.101602	0.026*	
H12B	0.131865	0.458418	0.062439	0.026*	
H12C	0.290992	0.488054	0.066435	0.026*	
C12	0.0820 (3)	0.0938 (3)	0.40960 (17)	0.0185 (8)	
H12D	0.046264	0.111991	0.457722	0.028*	
H12E	0.036883	0.026492	0.408038	0.028*	
H12F	0.061573	0.153370	0.364434	0.028*	
C21	0.8346 (3)	0.0792 (3)	−0.13645 (18)	0.0201 (7)	
H21A	0.814868	0.099776	−0.190980	0.030*	
H21B	0.749658	0.049098	−0.102401	0.030*	
H21C	0.908035	0.024203	−0.129171	0.030*	
C22	1.0399 (3)	0.2339 (3)	−0.18270 (17)	0.0236 (8)	
H22B	1.012324	0.243964	−0.235132	0.035*	
H22C	1.110033	0.176508	−0.172427	0.035*	
H22D	1.079343	0.302250	−0.179196	0.035*	
C31	0.2581 (3)	0.4638 (3)	0.56735 (19)	0.0268 (9)	
H31B	0.340628	0.417616	0.575523	0.040*	
H31C	0.177774	0.419428	0.564930	0.040*	
H31D	0.238166	0.496447	0.610681	0.040*	
C32	0.3259 (3)	0.4885 (3)	0.4126 (2)	0.0272 (9)	
H32B	0.401809	0.438141	0.429211	0.041*	
H32C	0.353752	0.536234	0.360159	0.041*	
H32D	0.241933	0.446834	0.411990	0.041*	

### Atomic displacement parameters (Å^2^)

**Table:**

	*U*^11^	*U*^22^	*U*^33^	*U*^12^	*U*^13^	*U*^23^
Te1	0.01126 (11)	0.00795 (11)	0.01136 (10)	−0.00191 (8)	−0.00056 (8)	−0.00204 (8)
Pd1	0.01032 (13)	0.00973 (13)	0.01451 (12)	−0.00091 (10)	−0.00135 (9)	−0.00456 (9)
Cl1	0.0150 (4)	0.0123 (4)	0.0193 (4)	−0.0034 (3)	−0.0003 (3)	−0.0048 (3)
S1	0.0130 (4)	0.0111 (4)	0.0155 (4)	0.0003 (3)	−0.0023 (3)	−0.0051 (3)
N1A	0.0113 (13)	0.0091 (14)	0.0141 (13)	0.0003 (11)	−0.0016 (10)	−0.0029 (10)
C1A	0.0155 (16)	0.0078 (16)	0.0141 (15)	−0.0024 (13)	−0.0049 (12)	−0.0005 (12)
O1	0.0134 (11)	0.0092 (11)	0.0084 (10)	0.0014 (9)	−0.0012 (8)	−0.0010 (8)
N1	0.0150 (14)	0.0093 (14)	0.0148 (13)	−0.0005 (11)	−0.0013 (10)	−0.0022 (11)
C1	0.0120 (16)	0.0085 (16)	0.0118 (15)	−0.0052 (13)	−0.0027 (12)	−0.0001 (12)
Te2	0.00944 (11)	0.00794 (11)	0.01094 (10)	−0.00101 (8)	−0.00156 (7)	−0.00269 (8)
Pd2	0.01263 (13)	0.01427 (14)	0.01753 (13)	−0.00153 (10)	−0.00298 (10)	−0.00488 (10)
S2	0.0148 (4)	0.0131 (4)	0.0168 (4)	−0.0020 (3)	−0.0023 (3)	−0.0045 (3)
N2	0.0128 (13)	0.0132 (15)	0.0145 (13)	0.0004 (12)	0.0020 (10)	−0.0006 (11)
O2	0.0143 (11)	0.0096 (12)	0.0093 (10)	0.0006 (9)	0.0004 (8)	−0.0015 (8)
N2A	0.0111 (13)	0.0137 (15)	0.0150 (13)	0.0021 (11)	−0.0043 (10)	−0.0048 (11)
C2A	0.0118 (16)	0.0097 (16)	0.0114 (15)	0.0010 (13)	−0.0060 (12)	0.0014 (12)
C2	0.0173 (17)	0.0128 (17)	0.0117 (15)	−0.0006 (14)	−0.0030 (12)	0.0007 (13)
Cl3	0.0163 (4)	0.0178 (4)	0.0163 (4)	−0.0028 (3)	−0.0007 (3)	−0.0077 (3)
O3	0.0186 (12)	0.0151 (13)	0.0205 (11)	0.0072 (10)	−0.0058 (9)	−0.0070 (9)
C3A	0.0197 (17)	0.0130 (18)	0.0162 (16)	−0.0029 (14)	−0.0094 (13)	−0.0033 (13)
C3	0.0284 (19)	0.0117 (17)	0.0137 (16)	−0.0002 (15)	−0.0073 (14)	−0.0035 (13)
Cl2	0.0188 (4)	0.0124 (4)	0.0234 (4)	−0.0047 (3)	−0.0064 (3)	−0.0025 (3)
Cl4	0.0210 (4)	0.0270 (5)	0.0217 (4)	−0.0029 (4)	0.0031 (3)	−0.0102 (4)
O4	0.0134 (11)	0.0158 (13)	0.0242 (12)	−0.0039 (10)	−0.0038 (9)	−0.0077 (10)
C4A	0.0141 (17)	0.0125 (18)	0.0245 (17)	−0.0067 (14)	−0.0114 (13)	0.0000 (14)
C4	0.0202 (18)	0.0156 (19)	0.0197 (17)	−0.0065 (14)	−0.0090 (14)	−0.0007 (14)
Cl5	0.0225 (5)	0.0388 (6)	0.0175 (4)	−0.0002 (4)	−0.0016 (3)	0.0015 (4)
C5	0.0111 (16)	0.0161 (18)	0.0188 (16)	−0.0045 (14)	−0.0037 (13)	−0.0002 (13)
C5A	0.0105 (16)	0.0129 (18)	0.0195 (16)	0.0009 (13)	−0.0055 (13)	0.0001 (13)
Cl6	0.0236 (5)	0.0292 (5)	0.0349 (5)	0.0099 (4)	−0.0122 (4)	−0.0126 (4)
C6A	0.0148 (16)	0.0090 (16)	0.0117 (15)	0.0000 (13)	−0.0053 (12)	0.0020 (12)
C6	0.0151 (17)	0.0118 (17)	0.0104 (15)	0.0000 (13)	−0.0021 (12)	0.0009 (12)
C7	0.0118 (16)	0.0155 (18)	0.0151 (16)	−0.0018 (14)	0.0000 (12)	−0.0019 (13)
C7A	0.0113 (16)	0.0111 (17)	0.0127 (15)	−0.0020 (13)	−0.0004 (12)	0.0006 (12)
C8A	0.0152 (16)	0.0101 (17)	0.0167 (16)	−0.0003 (13)	−0.0041 (13)	−0.0065 (13)
C8	0.0219 (18)	0.0112 (17)	0.0131 (15)	0.0023 (14)	0.0007 (13)	−0.0035 (13)
C9A	0.0168 (17)	0.0155 (18)	0.0188 (16)	−0.0036 (14)	0.0013 (13)	−0.0080 (14)
C9	0.0214 (18)	0.0132 (18)	0.0189 (17)	0.0041 (15)	−0.0033 (13)	0.0020 (13)
C10A	0.0162 (17)	0.0148 (18)	0.0147 (16)	−0.0010 (14)	−0.0005 (13)	−0.0004 (13)
C10	0.0157 (17)	0.0166 (18)	0.0188 (16)	−0.0011 (14)	0.0026 (13)	−0.0075 (14)
C11	0.0222 (18)	0.0190 (19)	0.0149 (16)	−0.0012 (15)	0.0013 (13)	0.0021 (14)
C11A	0.0130 (17)	0.021 (2)	0.0243 (17)	0.0042 (15)	−0.0003 (13)	−0.0117 (15)
C12A	0.0222 (18)	0.0097 (17)	0.0197 (17)	0.0032 (14)	−0.0062 (13)	−0.0029 (13)
C12	0.0132 (16)	0.0193 (19)	0.0200 (17)	−0.0016 (14)	0.0029 (13)	−0.0025 (14)
C21	0.0275 (19)	0.0146 (18)	0.0232 (18)	−0.0016 (15)	−0.0103 (14)	−0.0106 (14)
C22	0.0209 (18)	0.031 (2)	0.0174 (17)	−0.0031 (16)	0.0018 (14)	−0.0058 (15)
C31	0.0218 (19)	0.019 (2)	0.0300 (19)	−0.0012 (16)	−0.0004 (15)	0.0081 (15)
C32	0.024 (2)	0.028 (2)	0.042 (2)	0.0050 (17)	−0.0120 (16)	−0.0271 (18)

### Geometric parameters (Å, º)

**Table:**

Te1—O1	1.9836 (19)	C4—H4A	0.9500
Te1—O2	1.9871 (19)	C5—C6	1.389 (4)
Te1—C1	2.089 (3)	C5—H5A	0.9500
Te1—N2	2.450 (2)	C5A—C6A	1.375 (4)
Te1—N1	2.495 (2)	C5A—H5AA	0.9500
Te1—Te2	3.1222 (12)	C6A—C7A	1.506 (4)
Pd1—S1	2.2440 (9)	C6—C7	1.507 (4)
Pd1—Cl2	2.3049 (12)	C7—H7A	0.9900
Pd1—Cl1	2.3091 (11)	C7—H7B	0.9900
Pd1—Cl3	2.3278 (9)	C7A—H7AA	0.9900
S1—O3	1.477 (2)	C7A—H7AB	0.9900
S1—C21	1.768 (3)	C8A—H8AA	0.9900
S1—C22	1.777 (3)	C8A—H8AB	0.9900
N1A—C9A	1.471 (4)	C8—H8A	0.9900
N1A—C7A	1.477 (4)	C8—H8B	0.9900
N1A—C10A	1.483 (4)	C9A—H9AA	0.9800
C1A—C2A	1.384 (4)	C9A—H9AB	0.9800
C1A—C6A	1.407 (4)	C9A—H9AC	0.9800
Te2—O1	2.0071 (19)	C9—H9A	0.9800
Te2—O2	1.9844 (18)	C9—H9B	0.9800
Te2—N1A	2.477 (2)	C9—H9C	0.9800
Te2—N2A	2.459 (2)	C10A—H10A	0.9800
Te2—C1A	2.107 (3)	C10A—H10B	0.9800
N1—C10	1.471 (4)	C10A—H10C	0.9800
N1—C7	1.484 (4)	C10—H10D	0.9800
N1—C9	1.485 (4)	C10—H10E	0.9800
C1—C2	1.387 (4)	C10—H10F	0.9800
C1—C6	1.400 (4)	C11—H11A	0.9800
Pd2—S2	2.2444 (10)	C11—H11B	0.9800
Pd2—Cl5	2.2891 (11)	C11—H11C	0.9800
Pd2—Cl4	2.2997 (11)	C11A—H11D	0.9800
Pd2—Cl6	2.3194 (11)	C11A—H11E	0.9800
S2—O4	1.474 (2)	C11A—H11F	0.9800
S2—C32	1.769 (3)	C12A—H12A	0.9800
S2—C31	1.773 (3)	C12A—H12B	0.9800
N2—C11	1.476 (4)	C12A—H12C	0.9800
N2—C8	1.481 (4)	C12—H12D	0.9800
N2—C12	1.489 (4)	C12—H12E	0.9800
N2A—C11A	1.477 (4)	C12—H12F	0.9800
N2A—C8A	1.479 (4)	C21—H21A	0.9800
N2A—C12A	1.486 (4)	C21—H21B	0.9800
C2A—C3A	1.389 (4)	C21—H21C	0.9800
C2A—C8A	1.518 (4)	C22—H22B	0.9800
C2—C3	1.382 (5)	C22—H22C	0.9800
C2—C8	1.516 (4)	C22—H22D	0.9800
C3A—C4A	1.397 (4)	C31—H31B	0.9800
C3A—H3AA	0.9500	C31—H31C	0.9800
C3—C4	1.393 (4)	C31—H31D	0.9800
C3—H3A	0.9500	C32—H32B	0.9800
C4A—C5A	1.391 (4)	C32—H32C	0.9800
C4A—H4AA	0.9500	C32—H32D	0.9800
C4—C5	1.384 (4)		
			
O1—Te1—O2	76.92 (8)	C6—C5—H5A	120.0
O1—Te1—C1	96.99 (10)	C6A—C5A—C4A	120.5 (3)
O2—Te1—C1	93.43 (10)	C6A—C5A—H5AA	119.7
O1—Te1—N2	150.20 (8)	C4A—C5A—H5AA	119.7
O2—Te1—N2	75.53 (8)	C5A—C6A—C1A	118.4 (3)
C1—Te1—N2	73.66 (10)	C5A—C6A—C7A	123.1 (3)
O1—Te1—N1	77.12 (8)	C1A—C6A—C7A	118.5 (3)
O2—Te1—N1	148.63 (8)	C5—C6—C1	117.9 (3)
C1—Te1—N1	72.64 (10)	C5—C6—C7	123.2 (3)
N2—Te1—N1	124.18 (8)	C1—C6—C7	118.8 (3)
O1—Te1—Te2	38.79 (5)	N1—C7—C6	109.5 (2)
O2—Te1—Te2	38.14 (5)	N1—C7—H7A	109.8
C1—Te1—Te2	97.50 (8)	C6—C7—H7A	109.8
N2—Te1—Te2	113.10 (6)	N1—C7—H7B	109.8
N1—Te1—Te2	114.34 (6)	C6—C7—H7B	109.8
S1—Pd1—Cl2	88.13 (4)	H7A—C7—H7B	108.2
S1—Pd1—Cl1	91.84 (4)	N1A—C7A—C6A	110.0 (2)
Cl2—Pd1—Cl1	179.97 (3)	N1A—C7A—H7AA	109.7
S1—Pd1—Cl3	170.82 (3)	C6A—C7A—H7AA	109.7
Cl2—Pd1—Cl3	90.42 (4)	N1A—C7A—H7AB	109.7
Cl1—Pd1—Cl3	89.61 (4)	C6A—C7A—H7AB	109.7
O3—S1—C21	107.75 (14)	H7AA—C7A—H7AB	108.2
O3—S1—C22	109.19 (14)	N2A—C8A—C2A	109.6 (2)
C21—S1—C22	100.00 (17)	N2A—C8A—H8AA	109.7
O3—S1—Pd1	111.49 (9)	C2A—C8A—H8AA	109.7
C21—S1—Pd1	114.91 (11)	N2A—C8A—H8AB	109.7
C22—S1—Pd1	112.80 (12)	C2A—C8A—H8AB	109.7
C9A—N1A—C7A	112.2 (2)	H8AA—C8A—H8AB	108.2
C9A—N1A—C10A	109.0 (2)	N2—C8—C2	109.5 (2)
C7A—N1A—C10A	110.5 (2)	N2—C8—H8A	109.8
C9A—N1A—Te2	112.60 (18)	C2—C8—H8A	109.8
C7A—N1A—Te2	104.52 (16)	N2—C8—H8B	109.8
C10A—N1A—Te2	107.85 (16)	C2—C8—H8B	109.8
C2A—C1A—C6A	121.7 (3)	H8A—C8—H8B	108.2
C2A—C1A—Te2	119.5 (2)	N1A—C9A—H9AA	109.5
C6A—C1A—Te2	118.8 (2)	N1A—C9A—H9AB	109.5
Te1—O1—Te2	102.96 (8)	H9AA—C9A—H9AB	109.5
C10—N1—C7	111.5 (2)	N1A—C9A—H9AC	109.5
C10—N1—C9	109.8 (2)	H9AA—C9A—H9AC	109.5
C7—N1—C9	110.4 (2)	H9AB—C9A—H9AC	109.5
C10—N1—Te1	113.63 (17)	N1—C9—H9A	109.5
C7—N1—Te1	103.85 (17)	N1—C9—H9B	109.5
C9—N1—Te1	107.35 (16)	H9A—C9—H9B	109.5
C2—C1—C6	122.4 (3)	N1—C9—H9C	109.5
C2—C1—Te1	118.6 (2)	H9A—C9—H9C	109.5
C6—C1—Te1	119.0 (2)	H9B—C9—H9C	109.5
O2—Te2—O1	76.44 (8)	N1A—C10A—H10A	109.5
O2—Te2—C1A	95.66 (10)	N1A—C10A—H10B	109.5
O1—Te2—C1A	94.79 (10)	H10A—C10A—H10B	109.5
O2—Te2—N2A	76.19 (8)	N1A—C10A—H10C	109.5
O1—Te2—N2A	148.65 (8)	H10A—C10A—H10C	109.5
C1A—Te2—N2A	73.06 (10)	H10B—C10A—H10C	109.5
O2—Te2—N1A	148.72 (7)	N1—C10—H10D	109.5
O1—Te2—N1A	75.46 (8)	N1—C10—H10E	109.5
C1A—Te2—N1A	73.37 (10)	H10D—C10—H10E	109.5
N2A—Te2—N1A	125.35 (9)	N1—C10—H10F	109.5
O2—Te2—Te1	38.20 (6)	H10D—C10—H10F	109.5
O1—Te2—Te1	38.25 (5)	H10E—C10—H10F	109.5
C1A—Te2—Te1	97.51 (8)	N2—C11—H11A	109.5
N2A—Te2—Te1	113.27 (6)	N2—C11—H11B	109.5
N1A—Te2—Te1	112.83 (6)	H11A—C11—H11B	109.5
S2—Pd2—Cl5	86.92 (4)	N2—C11—H11C	109.5
S2—Pd2—Cl4	91.79 (4)	H11A—C11—H11C	109.5
Cl5—Pd2—Cl4	177.60 (3)	H11B—C11—H11C	109.5
S2—Pd2—Cl6	176.40 (3)	N2A—C11A—H11D	109.5
Cl5—Pd2—Cl6	89.84 (4)	N2A—C11A—H11E	109.5
Cl4—Pd2—Cl6	91.39 (4)	H11D—C11A—H11E	109.5
O4—S2—C32	108.18 (15)	N2A—C11A—H11F	109.5
O4—S2—C31	107.93 (15)	H11D—C11A—H11F	109.5
C32—S2—C31	99.92 (18)	H11E—C11A—H11F	109.5
O4—S2—Pd2	113.54 (9)	N2A—C12A—H12A	109.5
C32—S2—Pd2	111.49 (11)	N2A—C12A—H12B	109.5
C31—S2—Pd2	114.78 (11)	H12A—C12A—H12B	109.5
C11—N2—C8	111.0 (2)	N2A—C12A—H12C	109.5
C11—N2—C12	109.6 (2)	H12A—C12A—H12C	109.5
C8—N2—C12	112.5 (2)	H12B—C12A—H12C	109.5
C11—N2—Te1	107.77 (17)	N2—C12—H12D	109.5
C8—N2—Te1	104.65 (16)	N2—C12—H12E	109.5
C12—N2—Te1	111.16 (18)	H12D—C12—H12E	109.5
Te2—O2—Te1	103.65 (8)	N2—C12—H12F	109.5
C11A—N2A—C8A	110.7 (2)	H12D—C12—H12F	109.5
C11A—N2A—C12A	110.0 (2)	H12E—C12—H12F	109.5
C8A—N2A—C12A	111.2 (3)	S1—C21—H21A	109.5
C11A—N2A—Te2	115.2 (2)	S1—C21—H21B	109.5
C8A—N2A—Te2	105.52 (15)	H21A—C21—H21B	109.5
C12A—N2A—Te2	103.88 (17)	S1—C21—H21C	109.5
C1A—C2A—C3A	119.0 (3)	H21A—C21—H21C	109.5
C1A—C2A—C8A	118.5 (3)	H21B—C21—H21C	109.5
C3A—C2A—C8A	122.5 (3)	S1—C22—H22B	109.5
C3—C2—C1	118.5 (3)	S1—C22—H22C	109.5
C3—C2—C8	122.6 (3)	H22B—C22—H22C	109.5
C1—C2—C8	118.8 (3)	S1—C22—H22D	109.5
C2A—C3A—C4A	119.6 (3)	H22B—C22—H22D	109.5
C2A—C3A—H3AA	120.2	H22C—C22—H22D	109.5
C4A—C3A—H3AA	120.2	S2—C31—H31B	109.5
C2—C3—C4	119.9 (3)	S2—C31—H31C	109.5
C2—C3—H3A	120.0	H31B—C31—H31C	109.5
C4—C3—H3A	120.0	S2—C31—H31D	109.5
C5A—C4A—C3A	120.5 (3)	H31B—C31—H31D	109.5
C5A—C4A—H4AA	119.8	H31C—C31—H31D	109.5
C3A—C4A—H4AA	119.8	S2—C32—H32B	109.5
C5—C4—C3	120.9 (3)	S2—C32—H32C	109.5
C5—C4—H4A	119.5	H32B—C32—H32C	109.5
C3—C4—H4A	119.5	S2—C32—H32D	109.5
C4—C5—C6	120.0 (3)	H32B—C32—H32D	109.5
C4—C5—H5A	120.0	H32C—C32—H32D	109.5
			
C6A—C1A—C2A—C3A	−6.1 (4)	C2—C1—C6—C5	−5.3 (4)
Te2—C1A—C2A—C3A	176.0 (2)	Te1—C1—C6—C5	172.1 (2)
C6A—C1A—C2A—C8A	170.7 (3)	C2—C1—C6—C7	171.6 (3)
Te2—C1A—C2A—C8A	−7.1 (3)	Te1—C1—C6—C7	−11.0 (4)
C6—C1—C2—C3	4.0 (4)	C10—N1—C7—C6	161.0 (2)
Te1—C1—C2—C3	−173.4 (2)	C9—N1—C7—C6	−76.6 (3)
C6—C1—C2—C8	−173.5 (3)	Te1—N1—C7—C6	38.2 (2)
Te1—C1—C2—C8	9.1 (4)	C5—C6—C7—N1	153.4 (3)
C1A—C2A—C3A—C4A	2.8 (4)	C1—C6—C7—N1	−23.3 (4)
C8A—C2A—C3A—C4A	−173.9 (3)	C9A—N1A—C7A—C6A	−160.8 (2)
C1—C2—C3—C4	1.0 (4)	C10A—N1A—C7A—C6A	77.3 (3)
C8—C2—C3—C4	178.4 (3)	Te2—N1A—C7A—C6A	−38.5 (3)
C2A—C3A—C4A—C5A	1.7 (4)	C5A—C6A—C7A—N1A	−150.9 (3)
C2—C3—C4—C5	−4.5 (4)	C1A—C6A—C7A—N1A	26.6 (4)
C3—C4—C5—C6	3.1 (4)	C11A—N2A—C8A—C2A	162.0 (2)
C3A—C4A—C5A—C6A	−2.9 (5)	C12A—N2A—C8A—C2A	−75.3 (3)
C4A—C5A—C6A—C1A	−0.3 (4)	Te2—N2A—C8A—C2A	36.7 (3)
C4A—C5A—C6A—C7A	177.2 (3)	C1A—C2A—C8A—N2A	−23.8 (4)
C2A—C1A—C6A—C5A	4.9 (4)	C3A—C2A—C8A—N2A	153.0 (3)
Te2—C1A—C6A—C5A	−177.2 (2)	C11—N2—C8—C2	78.9 (3)
C2A—C1A—C6A—C7A	−172.8 (3)	C12—N2—C8—C2	−157.9 (2)
Te2—C1A—C6A—C7A	5.1 (3)	Te1—N2—C8—C2	−37.1 (2)
C4—C5—C6—C1	1.7 (4)	C3—C2—C8—N2	−154.2 (3)
C4—C5—C6—C7	−175.0 (3)	C1—C2—C8—N2	23.1 (4)

### Hydrogen-bond geometry (Å, º)

**Table:**

*D*—H···*A*	*D*—H	H···*A*	*D*···*A*	*D*—H···*A*
C7—H7*A*···Cl1	0.99	2.99	3.768 (3)	136
C7*A*—H7*AB*···Cl1	0.99	2.90	3.663 (3)	135
C9*A*—H9*AB*···Cl1^i^	0.98	2.85	3.697 (4)	145
C9*A*—H9*AC*···O1	0.98	2.49	3.000 (3)	112
C9—H9*B*···O4^ii^	0.98	2.64	3.355 (4)	130
C9—H9*B*···Cl5^ii^	0.98	2.87	3.741 (4)	149
C10*A*—H10*C*···Cl2^iii^	0.98	2.80	3.706 (3)	154
C10—H10*E*···O1	0.98	2.57	3.078 (4)	112
C10—H10*F*···Cl5^ii^	0.98	2.82	3.711 (4)	151
C11*A*—H11*E*···O2	0.98	2.56	3.048 (4)	110
C11*A*—H11*F*···Cl3^iii^	0.98	2.91	3.639 (4)	132
C12*A*—H12*A*···O3^iv^	0.98	2.42	3.316 (4)	151
C12*A*—H12*A*···Cl2^iv^	0.98	2.97	3.595 (3)	123
C12*A*—H12*B*···Cl2^iii^	0.98	2.80	3.678 (4)	150
C12—H12*D*···Cl6^v^	0.98	2.92	3.825 (3)	155
C12—H12*F*···O2	0.98	2.43	2.980 (4)	115
C21—H21*A*···Cl5^iv^	0.98	2.77	3.628 (3)	147
C21—H21*C*···Cl3^vi^	0.98	2.59	3.506 (3)	157
C22—H22*B*···Cl5^iv^	0.98	2.84	3.681 (4)	144
C22—H22*C*···Cl1^vi^	0.98	2.86	3.670 (4)	141
C22—H22*D*···Cl2	0.98	2.77	3.308 (4)	115
C31—H31*B*···O4^vii^	0.98	2.53	3.389 (4)	147
C31—H31*C*···Cl6^v^	0.98	2.94	3.796 (4)	146
C32—H32*B*···O4^vii^	0.98	2.46	3.336 (4)	149
C32—H32*C*···Cl5	0.98	2.73	3.275 (4)	115
C32—H32*D*···Cl4^v^	0.98	2.58	3.541 (4)	167

Symmetry codes: (i) −*x*+1, −*y*, −*z*; (ii) *x*, *y*−1, *z*; (iii) *x*−1, *y*, *z*; (iv) −*x*+1, −*y*+1, −*z*; (v) −*x*, −*y*+1, −*z*+1; (vi) −*x*+2, −*y*, −*z*; (vii) −*x*+1, −*y*+1, −*z*+1.
